# Prioritization, Identification, and Quantification of Emerging Contaminants in Recycled Textiles Using Non-Targeted and Suspect Screening Workflows by LC-ESI-HRMS

**DOI:** 10.1021/acs.analchem.4c02041

**Published:** 2024-08-20

**Authors:** Drew Szabo, Stellan Fischer, Aji P. Mathew, Anneli Kruve

**Affiliations:** † Department of Materials and Environmental Chemistry, Stockholm University, SE-106 91 Stockholm, Sweden; ‡ Swedish Chemicals Agency, SE-17267 Stockholm, Sweden; § Department of Environmental Science, Stockholm University, SE-106 91 Stockholm, Sweden

## Abstract

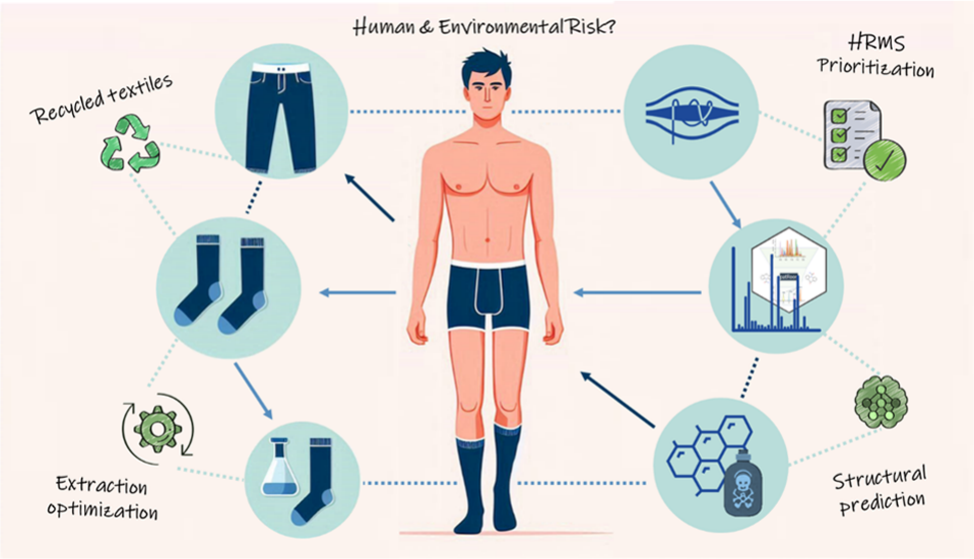

Recycled textiles are becoming widely available to consumers as manufacturers adopt circular economy principles to reduce the negative impact of garment production. Still, the quality of the source material directly impacts the final product, where the presence of harmful chemicals is of utmost concern. Here, we develop a risk-based suspect and non-targeted screening workflow for the detection, identification, and prioritization of the chemicals present in consumer-based recycled textile products after manufacture and transport. We apply the workflow to characterize 13 recycled textile products from major retail outlets in Sweden. Samples were extracted and analyzed by liquid chromatography coupled with high-resolution mass spectrometry (LC-HRMS). In positive and negative ionization mode, 20,119 LC-HRMS features were detected and screened against persistent, mobile, and toxic (PMT) as well as other textile-related chemicals. Six substances were matched with PMT substances that are regulated in the European Union (EU) with a Level 2/3 confidence. Forty-three substances were confidently matched with textile-related chemicals reported for use in Sweden. For estimating the relative priority score, aquatic toxicity and concentrations were predicted for 7416 features with tandem mass spectra (MS^2^) and used to rank the non-targeted features. The top 10 substances were evaluated due to elevated environmental risk linked to the recycling process and potential release at end-of-life.

## 1 Introduction

Societal pressure regarding unsustainable practices in the textile industry has recently led to the development of new manufacturing processes, including textile recycling that align more strongly with the United Nations Sustainable Development Goals. Today, textile garments can contain up to 98% recycled materials, thereby greatly reducing the environmental impact by increasing the life cycle of the materials.^1^ Many international authorities are implementing strategies to further improve the sustainability of the textile industry. For example, the European Commission aims to decrease the overall volume of textiles sent to landfill or incineration and will promote textile recycling programs.^2^ Despite obvious advantages, there are human and environmental hazards arising from the chemicals retained in the garment, which can transfer through the skin, or be released into the environment in the recycling process.^3^ The regulation of chemical risk from recycled textiles falls to existing legislation from their respective jurisdictions, such as the Swedish Chemicals Agency (Kemikalieinspektionen; KEMI), the European Chemical Agency (ECHA) and the USA Environmental Protection Agency (USEPA).

Chemicals used in recycled textiles can be derived from each of the major processing pathways: raw material, dyes, auxiliary and finishing substances common to all textile production and design; then the cleaning, separation, and preparation of recycled feedstock. Dyes are typically characterized by the chemistry of the chromophore,^4^ for example Azo dyes contain one or more double-bonded nitrogen groups (R_1_-N=N-R_2_),^5^ that can have deleterious impacts on receiving aquatic environments.^6^ Auxilliary substances include surfactants that are generally used throughout the textile manufacturing process and are discharged as effluent.^7^ Finishing chemicals used to enhance the utility and performance of textiles, such as per- and polyfluoroalkyl substances (PFASs) for water and oil repellency,^8^ and a range of brominated flame retardants (BRFs),^9^ also have known adverse impacts on human and environmental health.

Pretreatment of textiles intended for recycling often includes acid and alkaline washes to clean the textile fibers, and then oxidizers or enzymes can be used to dissolve or depolymerize cotton and polyester substrates.^10^ These processes act on the dyes, auxiliary, and finishing chemicals used in the production of the original textiles, leading to the transfer or transformation of these substances to the recycled textiles.

For the detection and identification of chemicals in textile related samples, sensitive analytical methods are essential. Chemicals from textile effluent are often detected in wastewater discharge using liquid chromatography coupled with high-resolution mass spectrometry (LC-HRMS).^11^ The primary focus has been on targeted or suspect screening of potentially harmful substances in textiles, particularly banned or restricted substances, as well as natural and synthetic dyes.^12^ These instruments are also capable of performing non-targeted screening, where all ionizable chemicals can be measured at once. However, this strategy often results in tens of thousands of chemicals from a single sample. Prioritization is required to filter and rank these chemicals to highlight chemicals of greater concern,^13^ particularly ones that have potential risk of adverse impacts to humans and the environment.^14^ Given the wide range of chemicals potentially present in recycled textiles, non-targeted screening is a promising strategy for detecting, identifying and quantifying the risk of chemicals in recycled textiles.

Previous studies have detected several banned and restricted substances such as quinolines, phthalates and nitroanilines at concentrations exceeding the threshold values in virgin textile materials in European markets.^15^ These substances can result in acute and chronic toxicity in human end-users,^16^ and can also lead to deleterious impacts on the environment, as many of the water-soluble chemicals used in textiles are then discharged to wastewater from laundry effluent.^17^

To date, there are limited empirical studies to determine if textiles produced from recycled materials can also contain these harmful substances.^3^ Therefore, this study aims to develop, validate, and apply a workflow for detecting, identifying as well as assessing the quantity and relative risk of chemicals in recycled textile products. For this purpose, a multiresidue extraction method was used on the textile samples to enable the detection of a wide chemical space using LC-HRMS. Then, suspect and non-targeted screening was performed, including the identification and prioritization of LC-HRMS features to highlight chemicals of concern based on their predicted ecotoxicity and concentrations. The proposed workflow was validated using a range of 38 chemicals with known structure where the accuracy of each process was evaluated. The developed workflow was then applied for the analysis of recycled textile items, including socks and underwear, purchased from Swedish retailers.

## 2 Experimental Section

### 2.1 Chemicals and Reagents

High-purity acetonitrile (75–05–8, >99.99%), methanol (67–56–1, >99.9%), and water (7732–18–5, >99%) were obtained from VWR International AB (Stockholm, Sweden). A QC mixture containing 41 chemicals was used for calibration standards, validation, and quality control, representing a wide range of chemical structures, ionization polarities, monoisotopic mass, and log*P*. From these, only 38 fell within the detectable MS^1^ range and will be reported (see Section 2.2). Detailed descriptions of each chemical, including their structural identifiers and predicted log*P* values can be found in the Supporting Information (Table S1). A ten-level calibration curve was prepared using these substances ranging in concentration from 1000 to 1 ng mL^–1^. Mass-labeled internal standards caffeine-^13^C_3_, haloperidol-*d*_4_, and imazalil-*d*_5_ were added to all calibration standards at a concentration of 50 ng mL^–1^.

### 2.2 Sample Characterization and Extraction

Textiles were purchased from various retailers in Stockholm, Sweden in August 2022 (Table 1). Underwear garments were selected for their increased relative risk of human dermal exposure, due to their long wear time and proximity to sensitive areas of the skin where perspiration is common. The individual items were selected based on a wide range of recycled material proportions.

**Table 1:** Overview of the Textile Samples Analyzed

	item	color	country	recycled material	recycled proportion (%)	other material	fabric density (g/m^2^)
1	underwear	black	Bangladesh	polyester	91	9% elastane	127.4
2	underwear	black	China	cotton	23	55% cotton 18% polyester 2% polyamide 2% elastane	174.1
3	underwear	white	China	cotton	23	55% cotton 18% polyester 2% polyamide 2% elastane	242.5
4	sock	black	China	polyester	98	2% elastane	205.0
5	sock	blue	China	polyester	98	2% elastane	212.2
6	sock	lavender	China	polyester	98	2% elastane	215.3
7	sock	purple	China	polyester	98	2% elastane	189.1
8	sock	white	China	polyester	98	2% elastane	222.0
9	underwear	black	Pakistan	polyamide	60	30% polyamide 10% elastane	250.4
10	underwear	beige	Pakistan	polyamide	60	30% polyamide 10% elastane	281.5
11	underwear	light green	Pakistan	polyamide	60	30% polyamide 10% elastane	225.3
12	underwear	olive	Pakistan	polyamide	60	30% polyamide 10% elastane	263.8
13	underwear	white	Pakistan	polyamide	60	30% polyamide 10% elastane	248.3

Triplicate textile samples (1 cm × 1 cm) were cut from each garment with a stainless steel scalpel by pressing the material with a sterile glass microscope slide to reduce contamination during handling. Each sample was weighed (±0.00001 g) and photographed on 0.5 mm graph paper to accurately determine the surface area with ImageJ according to the procedure described by Schneider et al.^18^

Textile samples were extracted using a multiresidue method where the solvents were selected to achieve the highest number of detectable features (Supporting Information Section 1.1). Briefly, samples were sonicated at 35–40 °C for 30 min with 5 mL 1:1 methanol/acetonitrile mixture in 15 mL glass vials. The supernatant was transferred to a clean 15 mL glass vial and the extraction was repeated on the textile samples. The pooled supernatant (10 mL) was evaporated to dryness at 40 °C under a gentle stream of nitrogen gas (>99.99%). The extract was reconstituted in 1 mL of 1:1 methanol/acetonitrile. For each batch of 11 samples, a method blank was obtained by repeating the extraction process with no textile. A 200 μL aliquot of each replicate extract was pooled and spiked with the QC mixture (100 ng mL^–1^) of known substances for quantitative analysis (Section 2.3).

### 2.3 LC-ESI-HRMS Acquisition

Liquid chromatography and electrospray ionization source conditions were previously described by Malm et al.^19^ Briefly, for positive ionization polarity, a 0.1% formic acid modifier was used for the aqueous phase (pH 2.7), while for negative ionization polarity, a 5 mM ammonium acetate buffer (pH 6.8) was used. In both polarities, 95% acetonitrile was used as the organic phase. Two microliters of the sample extracts as well as the calibration solutions were injected in a randomized order. The mobile phase flow rate was 0.350 mL min^–1^. The acquisition time was 25 min: the organic phase increased linearly from 5 to 100% over 20 min, then held at 100% for 5 min before returning to initial conditions at 25.1 min. The equilibration time between injections was 5 min. Separation was performed on a Phenomenex PS C18 column (150 mm × 1.8 mm, 2.6 μm), held at 40 °C, for each positive and negative polarity mode.

Compounds were ionized by electrospray ionization (ESI) coupled to a QExactive HF Orbitrap (ThermoFisher Scientific) to acquire tandem mass spectra in data-dependent acquisition mode. Precursor ions (MS^1^) in the 100–1500 *m*/*z* range were measured with 60,000 unit resolution. An inclusion list was used with a quadrupole window of 0.4 Da were used the acquire tandem mass spectra (MS^2^) with normalized collision experiments of 30, 70, and 120 V respectively at 30,000 unit resolution. For scans in which no *m*/*z* from the inclusion list are detected, the five most abundant peaks were selected by the instrument for MS^2^ analysis. The inclusion list was based on the Swedish Chemical Agency (KEMI) Market List,^20^ for chemicals that can be expected to occur on the EU market, including raw chemicals imported or produced in Sweden. Substances were manually curated by filtering for chemicals with associated uses in the textile industry and other textile-related chemicals known to be used in the EU. A final list of 1552 substances, their identifying information, and the exposure score, can be found in the Supporting Information (Table S2).

### 2.4 Data Analysis

#### 2.4.1 Peak Picking and Alignment

Data analysis was performed using patRoon v2.3^21^ in RStudio v2021.09.0 and R v4.2.2 with all relevant dependencies installed. Features from each sample were extracted and aligned using XCMS,^22^ optimized based on the extraction yield of spiked chemicals in a calibration mixture. Features were initially filtered for intensity threshold (positive: 100,000; negative: 50,000 units) and for presence in each of the triplicate analyses within 50% relative standard deviation. Average group peak intensities must also exceed the average method blank peak intensity by a factor of 3 to eliminate false positives.^23^ The MS^1^ extracted ion chromatogram (EIC) quality for each aligned feature group was evaluated with NeatMS, where groups including one or more “high quality” peaks were included for further analysis.^24^ Componentization was performed using CAMERA, where only [M + H]^+^ and [M – H]^−^ adducts were preserved in positive and negative modes respectively.^25^ MS^2^ peak lists were then generated using the mzR package, using a precursor *m*/*z* window of 0.4 Da, a retention time window of 5 s, and filtered to include peaks with MS^2^ and >5% relative average peak intensity from all collision energies (Table S10).

#### 2.4.2 Structural Annotation

Formulas were generated for all features using SIRIUS CSI:FingerID v5.8.4 for [M + H]^+^ and [M – H]^−^ adduct formation and including the following elements: C, H, O, N, P, S, F, Cl, Br.^26^ All features were then compared with the MassBank v2022.06^27^ library with cosine similarity score >0.5. Simultaneously, SIRIUS and MetFrag v2.5.0^28^ were used for tentative structural assignment for features with MS^2^ spectra using forward and inverse *in silico* approaches, respectively. A complete list of feature finding and structural annotation parameters can be found in Table S11. Compound identification results from SIRIUS, MetFrag and MassBank were evaluated to meet two criteria: (1) the annotation with the highest score is used, (2) the annotation matches >2 MS^2^ peaks, and for in silico matches (3) the Tanimoto similarity between predicted structures is >0.8. Confidence in chemical annotation was assessed according to Schymanski et al.:^29^ where local spectral library matches (including retention time) were ranked Level 1, online spectral library matches were ranked Level 2b, confident *in silico* predictions were ranked Level 3, and unequivocal formula predictions were ranked Level 4. No local library matches were attempted and the highest level reported in this study is 2b.

#### 2.4.3 Suspect Screening and Retention Time Index Calculation

Suspect screening was performed against two lists derived from the NORMAN Suspect List Exchange: (1) 1522 substances identified as textile-related chemicals from the KEMI Market List–S17; and (2) 340 substances identified as persistent, mobile, and toxic (PMT) from the REACH legislation–S36.^30^ The *m*/*z* from the measured MS^1^ was matched to each list (±2 mDa) for all calculated [M + H]^+^ and [M – H]^−^ adducts. Retention time indices (RTI) from calibrants in the QC mixture were plotted against measured retention times (*R*^2^ = 0.9275). This linear regression model was used to predict the retention time of each suspect chemical based on the RTI predictions described by Aalizadeh et al.^31^ Suspect chemicals were filtered by allowing a maximum deviation of ±120 s (RTI: ±55.99) from the predicted retention time.

#### 2.4.4 Quantification

MS2Quant was used via patRoon to predict the concentration of each of the non-targeted features detected in recycled textile samples based on the MS^2^ spectra.^32^ First, the integrated peak areas from 25 calibration chemicals detected at a minimum of 5 levels were used to calculate the response factors (Table S8). The response factors of these chemicals were then used further to transfer the log* IE* predictions from the MS2Quant *xgbTree* algorithm to instrument-specific response factors for the non-targeted features with SIRIUS fingerprints. Finally, integrated peak areas and instrument-specific predicted response factors were used to estimate the concentration of the non-targeted features in the textile extracts.

#### 2.4.5 Toxicity and Priority Scoring

MS2Tox (via patRoon) was used to predict the aquatic toxicity for each non-targeted feature detected in recycled textile samples.^33^ Here, calculated SIRIUS fingerprints for each unknown feature are processed with a model previously trained for predicting aquatic LC_50_ in fish. A priority score was then assigned to each unknown feature based on the predicted hazard quotient
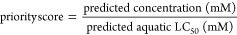


Due to the uncertainty in the concentration and toxicity predictions, the priority score is only used to compare features to one another, rather than accurate quantitative hazard or risk. Code used to perform data analysis reported in this study is available from https://github.com/kruvelab/ReStart.

#### 2.4.6 Workflow Validation

The acquisition, detection, extraction and structural identification workflow was validated by evaluating the performance of 38 chemicals added to pooled samples from the QC mixture. First, the number of chemicals ionized in positive and negative mode, and had MS^2^ triggered, were matched with the list of chemicals from the QC mixture within 2 mDa. Then, the structural annotation for each feature with acquired MS^2^ was evaluated by calculating the true positive rate (TPR), precision and F1 score using the following equations^34^
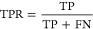

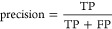

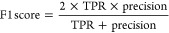
where true positives (TP) equals the number of features with correctly annotated structure, false negatives (FN) equals the number of features where the chemical was incorrectly annotated or left unannotated, and false positives (FP) equals the number of features annotated incorrectly, excluding features not annotated. The F1 score provides an equally scaled evaluation of both the TPR and precision.

## 3 Results and Discussion

### 3.1 Validation and Annotation Performance

From the 38 chemicals in the QC mixture added to the pooled samples, 29 unique chemicals were matched with 43 LC-HRMS features in positive and negative ionization modes using LC-ESI-HRMS within 5 mDa mass accuracy (Table S3). From the nine chemicals from the QC mixture that were not matched, three were molecular ions [M]^+^, which are not currently supported in this workflow (chlormequat, tetraethylammonium, and tetrahexylammonium). The low concentration of the QC mixture in the pooled samples (100 ng mL^–1^) and poor ionization efficiency of a further six chemicals may have suppressed ionization and detection of MS^1^ (Table S1). MS^2^ was triggered for 28 positive and negative mode features representing 23 unique chemicals, limiting the overall structural annotation. Compounds ranging in mass from uracil (monoisotopic mass = 112.0273 Da) to tylosin (monoisotopic mass = 915.5192 Da) were detected with mass accuracies within 6.5 ppm (maximum absolute error = 1.92 mDa), except for TCMTB (20.9 ppm; 4.99 mDa) and butocarboxim (21.1 ppm; 4.03 mDa). The annotated chemicals ranged in log*P* from −2.7 (aspartame) to 6.9 (rifaximin), indicating that the workflow is applicable for chemicals with a wide mass and polarity range.

*In silico* structural annotation of the 21 features with MS^2^ spectra from the QC mixture yielded 89 and 72% correct structural annotation with the highest rank by MetFrag and SIRIUS in positive mode, respectively. In negative mode, MetFrag and SIRIUS correctly annotated with the highest rank 100 and 83% of the 7 features with MS^2^, respectively. The performance was comparable in true positive rate and precision to previous studies evaluating MetFrag and SIRIUS.

Features with the highest-ranked structure annotation by MassBank were 100% accurate in both positive and negative ionization modes, despite fewer overall annotations compared to *in silico* methods (Table 2). All of the chemicals spiked into the calibration solutions have entries in the MassBank database with high-quality tandem mass spectra, except for Ivermectin B1a (CASRN: 70288–86–7), resulting in an overall TPR of 67% in positive mode and 57% in negative mode.

**Table 2:** Summary of Ionization, Detection, Acquisition and Structural Annotation Performance from Chemicals Added to Pooled Samples^35^

	positive			negative		
spiked	38			38		
LC-ESI^a^	34			14		
detected MS1	32			11		
acquired MS2	21			7		
	*MetFrag*	*SIRIUS*	*MassBank*	*MetFrag*	*SIRIUS*	*MassBank*
correct	17	13	14	6	5	4
TPR	0.81	0.62	0.67	0.86	0.71	0.57
precision	0.89	0.72	1.00	1.00	0.83	1.00
F1 score	0.85	0.67	0.80	0.92	0.77	0.73

a LC-ESI amenability based on empirical spectral information collated by PubChem.^35^

The number of features with MS^2^ acquired from pooled recycled textile extracts was a known limitation of the DDA acquisition selected for this methodology. Almost all of the precursors were observed in the MS^1^ (Table 2); however, the low response for many chemicals at 100 ng mL^–1^ excluded the acquisition of MS^2^ for one-third of features. Since spectral library and *in silico* annotation are dependent on high-quality MS^2^ to make predictions, highly confident structural annotations were not possible. Despite this limitation, annotation performance results from features with MS^2^ agree with previous assessments of the library and *in silico* results.

### 3.2 Summary of Features

The total number of aligned features extracted from all samples and blanks was 114,965 in positive mode and 53,068 in negative mode. In positive mode, 25,353 (22%) features remained after initial threshold filtering; 17,314 (15%) features remained after EIC peak evaluation; 16,027 (14%) features with predicted [M + H]^+^ ion via componentization, and 5999 (0.1%) features had acquired MS^2^. In negative mode, 7711 (15%) features remained after initial threshold filtering; 4980 (9%) remained after EIC peak evaluation; 4092 (8%) features with predicted [M – H]^−^ adduct formation; and 1417 (3%) features had acquired MS^2^.

An average of 2722 positive mode features were detected in each sample, ranging between 220 (Sample 6) and 4461 (Sample 11). The majority of features detected from recycled textiles were found only in one sample (70%). Then the proportion of features detected drops from 9% in two samples to <0.1% of features detected in all 13 samples. An average of 515 negative mode features were detected in each sample, ranging between 48 (Sample 6) and 1605 (Sample 9). 84% of features were only found in one sample, then also falls from two samples (9%) to all 13 samples (<0.1%).

### 3.3 Suspect Screening

#### 3.3.1 Persistent, Mobile, and Toxic Chemicals (REACH)

A total of 59 non-targeted features from recycled textile samples were matched with 65 unique chemicals from the REACH PMT list based on the *m*/*z*. Two positive mode features and four negative mode features were matched with more than one chemical in the REACH PMT list. Nevertheless, the predicted RTI was used to eliminate false-positive candidates in both positive and negative modes, yielding 7/26 and 19/39 features within the predicted retention time range, respectively (Table S4).

The tandem mass spectra from six positive and negative mode features were matched with either the MassBank spectral library or *in silico* fragments, resulting in structural annotation confidence of Level 2 and Level 3 (Table 3). Trifluoromethanesulfonic acid, 2,4-dinitrophenol, 4-aminotoluene-3-sulfonic acid, dibutyl hydrogen phosphate, and dinoseb were each detected in negative ionization mode, and 2,2′-dimorpholinyldiethyl ether was detected in positive ionization mode. 2,4-dinitrophenol and dibutyl hydrogen phosphate, which were detected in 46 and 62% of samples respectively, had a higher relative response in recycled polyester textiles compared with recycled cotton and recycled polyamide samples (Table S4). Dinoseb, detected in 69% of samples, was found in all sample types with no clear trend in relative response. Similarly, the low detection frequency of trifluoromethanesulfonic acid, 4-aminotoluene-3-sulfonic acid, and 2,2′-dimorpholinyldiethyl ether did not explain trends among sample groups.

**Table 3:** List of Features Extracted from Recycled Textile Samples (*n* = 13) and Matched with the REACH PMT and KEMI Textile-Related Suspect Lists (±2 mDa) in Positive and Negative Ionization Modes^a^

#	name	formula	exact mass	RT (min)	adduct	detection frequency (%)	mass error (ppm)	conf.	exp.
REACH Persistent, Mobile, and Toxic (PMT)									
1	trifluoromethanesulfonic acid	CHF_3_O_3_S	149.9598	1.24	[M – H]^−^	23	–0.8	2b	NA
2	2,4-dinitrophenol	C_6_H_4_N_2_O_5_	184.0120	3.24	[M – H]–	46	0.3	2b	NA
3	4-aminotoluene-3-sulfonic acid	C_7_H_9_NO_3_S	187.0303	1.94	[M + Cl]^−^	8	–0.9	2b	NA
4	dibutyl hydrogen phosphate	C_8_H_19_O_4_P	210.1021	4.81	[M – H]^−^	62	0.8	3	NA
5	dinoseb	C_10_H_12_N_2_O_5_	240.0746	7.74	[M – H]^−^	69	1.3	3	NA
6	2,2′-dimorpholinyldiethyl ether	C_12_H_24_N_2_O_3_	244.1787	2.74	[M + H]^+^	23	1.7	2b	NA
KEMI Textile-Related Substances									
1	cyclopentasiloxane, 2,2,4,4,6,6,8,8,10,10-decamethyl-	C_10_H_30_O_5_Si_5_	370.0940	19.16	[M + H]^+^	46	0.64	3	25
2	benzenesulfonic acid, 4-methyl-	C_7_H_8_O_3_S	172.0194	2.21	[M – H]^−^	31	0.60	2b	24
3	1-tetradecanamine, *N*,*N*-dimethyl-, *N*-oxide	C_16_H_35_NO	257.2719	12.83	[M + H]^+^	62	0.93	3	23
4	ethanol, 2-[2-[2-(dodecyloxy)ethoxy]ethoxy]-	C_18_H_38_O_4_	318.2770	15.54	[M + H]^+^	54	0.25	3	22
5	3,6,9,12-tetraoxatetracosan-1-ol	C_20_H_42_O_5_	418.0954	15.32	[M + H]^+^	31	0.51	2b	22
6	anthra[2,1,9-def:6,5,10-*d*′*e*′*f*′]diisoquinoline-1,3,8,10(2*H*,9*H*)-tetrone, 2,9-dimethyl-	C_26_H_14_N_2_O_4_	362.3032	12.73	[M + H]^+^	8	–3.65	3	22
7	l-histidine	C_6_H_9_N_3_O_2_	155.0695	4.57	[M + H]^+^	23	–1.23	3	20
8	dodecanamide, *N*,*N*-bis(2-hydroxyethyl)-	C_16_H_33_NO_3_	287.2460	10.79	[M + H]^+^	77	1.98	3	20
9	dodecanamide, *N*,*N*-bis(2-hydroxyethyl)-	C_16_H_33_NO_3_	327.3137	12.36	[M + H]^+^	54	0.43	2b	20
10	octadecanamide, *N*-(2-hydroxyethyl)-	C_20_H_41_NO_2_	287.2460	13.86	[M + H]^+^	8	0.56	3	20
...									
34	1*H*-Imidazole-1-ethanol, 2-heptadecyl-4,5-dihydro-	C_22_H_44_N_2_O	352.3454	12.94	[M + H]^+^	8	0.85	3	11
35	benzonitrile, 2-[2-[4-[(2-cyanoethyl)ethylamino]phenyl]diazenyl]-5-nitro-	C_18_H_16_N_6_O_2_	348.1335	12.52	[M + H]^+^	8	–0.57	3	10
36	2*H*-1-benzopyran-2-one, 3-(1*H*-benzimidazol-2-yl)-7-(diethylamino)-	C_20_H_19_N_3_O_2_	333.1477	8.85	[M + H]^+^	23	0.65	3	7
37	benzenesulfonic acid, 5-amino-2-methyl-	C_7_H_9_NO_3_S	187.0303	1.94	[M + Cl]^−^	8	–0.94	3	6
38	benzenemethanamine, *N*-(phenylmethyl)-	C_14_H_15_N	197.1204	5.42	[M + H]^+^	15	0.73	3	6
39	decanamide, *N*,*N*-bis(2-hydroxyethyl)-	C_14_H_29_NO_3_	259.2147	10.17	[M + H]^+^	54	0.71	3	4
40	benzenamine, 2-chloro-4,6-dinitro-	C_6_H_4_ClN_3_O_4_	216.9890	9.49	[M – H]^−^	23	0.87	3	3
41	benzaldehyde, 3,5-bis(1,1-dimethylethyl)-4-hydroxy-	C_15_H_22_O_2_	234.1620	14.24	[M + H]^+^	85	0.28	2b	3
42	acetamide, *N*-[2-[2-(3-chloro-4-nitrophenyl)diazenyl]-5-[[2-(2,5-dioxo-1-pyrrolidinyl)ethyl]ethylamino]phenyl]-	C_22_H_23_ClN_6_O_5_	486.1418	11.14	[M – H]^−^	15	1.22	3	2
43	propanenitrile, 3-[[4-[2-(2,6-dichloro-4-nitrophenyl)diazenyl]phenyl](2-hydroxyethyl)amino]-	C_17_H_15_Cl_2_N_5_O_3_	407.0552	12.95	[M + H]^+^	15	1.59	3	2

a Conf. indicates the structural annotation confidence level as described by Schymanski et al. Exp. indicates the exposure score used to estimate patterns and volume of use in Sweden and the EU.

2,4-dinitrophenol and 4-aminotoluene-3-sulfonic acid each have reported industrial uses in the manufacturing of textile dyes and pigments. 2,2′-dimorpholinyldiethyl ether is reported uses as a catalyst and intermediate for foams and adhesives,^36^ and since its inclusion to the REACH PMT list, has been detected in surface waters^37^ and wastewater effluent.^38^ Trifluoromethanesulfonic acid, an ultrashort chain PFAS, is commonly used for esterification, rearrangements, and polymerization in chemical processes.^36^ Dinoseb and dibutyl hydrogen phosphate, annotated with a Level 3 confidence, are also chemicals potentially used in the textile finishing process, as a pesticide^39^ and flame-retardant,^40^ respectively (Table 3). The presence of each of these chemicals in some, but not all, of the recycled textile samples (detection frequency: 8–69%) suggests that these substances may not be used ubiquitously in the production of the textiles. Rather, the sources may be from heterogeneous contaminated feedstock, or introduced throughout the packing and distribution phase. Each of these chemicals has restricted use in the European Union due to their persistence, mobility, and toxicity, limiting their concentration in these textiles to 0.1% of the total garment by weight. In follow-up studies, the concentration of each of these chemicals should be determined with reference analytical standards to check compliance with these regulations. Nevertheless, extended exposure to these chemicals from underwear should be avoided to reduce the risk of adverse impacts, especially to vulnerable end users.

#### 3.3.2 Textile-Related Substances

A total of 403 features detected in recycled textile samples were matched with 340 unique chemicals from the KEMI textile-related substance list; in positive mode and negative mode, 26 and 35 features returned more than one match to the list respectively. Therefore, the predicted RTI for each of the chemicals in the suspect list was used to eliminate false-positive candidates, resulting in 108/216 and 92/187 LC-HRMS features with candidate structure in positive and negative modes (Table S5). Forty-three features were matched with MassBank spectral library (Level 2b) and *in silico* (Level 3) structural annotations combined in positive and negative ESI mode. The features with the highest exposure score include common surfactants used in high volume throughout textile manufacturing (Table 4), including several amines, alcohols, and glycols. These chemical groups are associated with skin irritation and environmental hazards that will have adverse impacts to human and environmental health at high concentrations. Features with the lowest exposure score tend to include auxiliary and finishing chemicals such as pesticides (Table 4).

**Table 4:** List of Top 10 Features Detected in Positive Ionization Mode from Recycled Textile Samples (*n* = 13), with Positive Library or *In Silico* Structural Annotation, Ranked in Order of Decreasing Priority Score^a^

rank	name	formula	*m*/*z*	adduct	RT (min)	det freq (%)	conf.	LC_50_ (mM)	priority score
1	3,6,9,12-tetraoxatetracosan-1-ol	C_20_H_42_O_5_	363.3104	[M + H]^+^	15.98	38	3	0.25	1.55
2	laureth-3	C_18_H_38_O_4_	319.2844	[M + H]^+^	15.54	54	3	0.37	0.94
3	3,6,9,12-tetraoxatetracosan-1-ol	C_20_H_42_O_5_	363.3104	[M + H]^+^	16.82	38	3	0.25	0.80
4	laureth-3	C_18_H_38_O_4_	319.2843	[M + H]^+^	16.80	46	3	0.37	0.56
5	PA(P-18:0/22:6(4*Z*,7*Z*,10*Z*,13*Z*,16*Z*,19*Z*))	C_43_H_73_O_7_P	733.5187	[M + H]^+^	19.67	15	3	0.41	0.46
6	tris(2-ethylhexl)phosphate	C_24_H_51_O_4_P	435.3681	[M + H]^+^	17.60	31	2b	0.26	0.27
7	PA(22:0/22:6)	C_47_H_81_O_8_P	805.5763	[M + H]^+^	19.64	23	3	0.57	0.23
8	PA(P-18:0/22:6(4*Z*,7*Z*,10*Z*,13*Z*,16*Z*,19*Z*))	C_43_H_73_O_7_P	733.5168	[M + H]^+^	18.36	46	3	0.26	0.22
9	myreth-3	C_20_H_42_O_4_	347.3156	[M + H]^+^	18.85	46	3	0.32	0.21
10	PA(O-16:0/18:4(6*Z*,9*Z*,12*Z*,15*Z*))	C_37_H_67_O_7_P	655.4681	[M + H]^+^	18.44	46	3	0.18	0.14

a Priority score = predicted concentration/predicted toxicity.

Some chemicals common to both the REACH PMT and KEMI screening lists were matched with the same features in both processes, such as 2,4-dinitrophenol and 4-aminotoluene-3-sulfonic acid (Table S5). However, the feature identified as dinoseb in the PMT screening (Level 3), was more confidently annotated as its isomer, dinoterb, in the KEMI screening (Level 2b). Perhaps due to their isomeric structure, both dinoseb and dinoterb have reported reproductive toxicity and therefore have restricted usage in the EU. Dinoterb is a registered substance in the EU REACH framework and is listed as a known reproductive toxicant, however, it is absent from the PMT list due to relatively little persistence and mobility data available at the current time. Nevertheless, its use is banned in cosmetics and limited as a residual pesticide on food crops. The high detection frequency of dinoterb from recycled textiles suggests that this is a widely used finishing substance for pest control for textiles, despite its regulation in Europe and the USA.

#### 3.3.3 Non-Targeted Screening

A total of 591 features in positive mode had the Priority Score calculated, based on the predicted aquatic toxicity (mM) and concentration (mM), ranging from 5 × 10^0^ to 4 × 10^–6^ units. Predicted aquatic toxicity for all features ranged from 1.99 mM (salicylic acid; C_7_H_6_O_3_) to 0.03 mM (oleamidopropyl dimethylamine; C_23_H_46_N_2_O); however, these values are indicative and only used in combination with the maximum predicted concentration to rank features (Table S6). The top 10 features based on Priority Score and confident structural annotation yielded LC_50_ of 0.14 mM or lower (Table 5).

**Table 5:** List of Top 10 Features Detected in Negative Ionization Mode from Recycled Textile Samples (*n* = 13), with Positive Library or *In Silico* Structural Annotation, Ranked in Order of Decreasing Predicted Aquatic Toxicity

name	formula	*m*/*z*	adduct	RT (min)	det freq (%)	confidence	LC_50_ (mM)
antioxidant 1790	C_42_H_57_N_3_O_6_	698.4179	[M – H]^−^	19.39	15	3	0.05
dinoterb	C_10_H_12_N_2_O_5_	239.0677	[M – H]^−^	7.74	69	2b	0.07
antioxidant 245	C_34_H_50_O_8_	585.3438	[M – H]^−^	16.42	38	3	0.08
quercetin 3,7-diglucuronide	C_27_H_26_O_19_	653.0962	[M – H]^−^	10.19	8	3	0.09
5-chloro-7-nitroquinolin-8-ol	C_9_H_5_ClN_2_O_3_	222.9920	[M – H]^−^	7.77	15	3	0.09
2,6-dinitro-4-chlorophenol	C_6_H_3_ClN_2_O_5_	216.9661	[M – H]^−^	5.22	15	3	0.13
2-hexadecylbenzenesulfonate	C_22_H_38_O_3_S	381.2477	[M – H]^−^	12.98	31	3	0.13
taxifolin	C_15_H_12_O_7_	301.0513	[M – H]^−^	6.49	8	2b	0.13
canrenone	C_22_H_28_O_3_	339.2005	[M – H]^−^	21.45	8	3	0.14
canrenone	C_22_H_28_O_3_	339.2004	[M – H]^−^	12.43	38	3	0.14

Only one feature from the top 10 ranked substances was annotated with Level 2 confidence: tris(2-ethylhexl)phosphate (TEHP, cosine similarity of spectral matching = 0.51). TEHP, like other organophosphate esters (OPEs), is an industrial chemical primarily used as a plasticizer and flame retardant. While OPEs in general are widely cited for use in textiles, TEHP has only been detected via proxy media, such as particulate matter,^41^ natural waters,^42^ and wastewater^43^ associated with textile facilities. To the authors’ knowledge, TEHP has previously not been directly observed from textile or recycled textile extracts. TEHP has been suspected to be an endocrine-disrupting chemical^44^ and a systematic review has revealed a low but significant carcinogenic risk from airborne exposure.^45^ The absence of TEHP from the REACH PMT and curated KEMI suspect screening lists indicates that this substance is currently not perceived to be a human health risk from textile manufacturing; however, its high priority score from this study warrants further investigation into the exposure to this chemical from textiles.

Laureth-4 (3,6,9,12-Tetraoxatetracosan-1-ol) had the highest priority score (1.55) and was positively matched with MassBank for two closely eluting LC/HRMS features (RT = 15.98 and 16.82), possibly indicating the presence of branched and linear isomers, and decreasing the confidence in structural annotation. Laureth-4 and laureth-3 are common surfactants that were tentatively identified by the KEMI textile-related suspect screening list (see above), indicating a known use in textile-related manufacturing. Similarly, myreth-3 has reported uses as a surfactant and emulsifier in consumer products, though mainly cosmetics. At high concentrations, these substances are skin and eye irritants for humans, which may be contributing to the high aquatic toxicity predicted by the MS2Tox model and increasing the Priority Score for these chemicals. The downstream impacts of these substances from laundry discharge may pose a risk to receiving environments; however, these types of substances are also easily degradable and form transformation products in most wastewater treatment processes.^46^

Three phosphatidic acids (PAs) were also ranked highly according to the Priority Score, combining predicted toxicity and concentration. In general, PAs are used in the bleaching and dying processes as they form a liposome around the hydrophobic dye chemicals and are used to dispense them to the textile fibers.^47^ This process is regarded as a “green” process as the lipids can then be safely diverted to wastewater streams where the chemicals are readily degraded.^48^ To the authors’ knowledge, there is limited toxicity information regarding these PAs to support the value predicted by the MS2Tox model. Nevertheless, other surfactants are known to have adverse impacts on human and environmental health. Since these chemicals are widely used in the textile industry, the concentration and associated risk of PAs should be investigated further.

A total of 177 LC-HRMS features in ESI negative mode had the toxicity predicted ranging between tris(4-*tert*-butyl-3-hydroxy-2,6-dimethylbenzyl) isocyanurate (C_42_H_57_N_3_O_6_, 0.03 mM) and 3,5-dioxohexanoic acid (C_6_H_8_O_4_, 1.06 mM). Concentrations were not calculated for features acquired in negative ionization mode due to limitations with the version of MS2Quant at the time of publication. Therefore, the top 10 features ranked highest in aquatic toxicity that have confident structural annotation are each <0.14 mM (Table S7).

Dinoterb, which was positively matched with MassBank, was ranked second highest in predicted toxicity, in addition to its presence in the KEMI textile-related suspect screening list. Pesticides like dinoterb were present in the MS2Tox training set,^33^ due to the widely available information on the aquatic toxicity in fish, which lends confidence to its prioritization as a chemical of interest in this study.

Taxifolin was also positively matched with MassBank (cosine similarity = 0.89) but only detected in a single recycled textile sample. Taxifolin is a plant derivative, reportedly used for its antioxidant and anti-inflammatory properties^49^ but flavonoids similar to taxifolin have also been found to have antimicrobial action.^50^ Formulations containing taxifolin and other flavonoids are contained in mixtures that are applied to textiles in the finishing process to help prevent bacterial growth.^51^ Similarly, antioxidant 1790, antioxidant 245, quercetin 3,7-diglucuronide, and canrenone were predicted to have high aquatic toxicity, albeit with lower structural confidence. However, human exposure to these chemicals is of lesser concern because of their use as therapeutic pharmaceuticals, which, due to the high biological activity, may have fingerprints similar to chemicals of higher toxicity. Furthermore, the antioxidants have a neutral mass >500 Da, limiting their dermal exposure pathways from the use of recycled textiles, but may present a risk to receiving environments from the discharge of laundry effluent.

The next features with the highest predicted toxicity were 5-chloro-7-nitroquinolin-8-ol and 2,6-dinitro-4-chlorophenol. As chlorinated nitrophenolic chemicals, this group of substances are known for their use in the synthesis of dyes and pesticides and are likely present in recycled textiles as unsynthesized reagents. Unfortunately, very limited information is available regarding specific use cases or toxicity for either of these chemicals. The high predicted toxicity is likely derived from similarity to other nitroaromatic chemicals^52^ and chlorinated substances.^53^

In one-third of the recycled textile samples, 2-hexadecylbenzenesulfonate was detected and had a relatively high predicted aquatic toxicity. There are limited reported uses for this chemical, while other shorter-chain benzenesulfonic acids are used as surfactants and cleaning agents for industrial applications. There is also no toxicity information available for 2-hexadecylbenzenesulfonate; however, the shorter-chain benzenesulfonic acids also have reported acute toxicity and skin sensitization properties.

#### 3.3.4 Comparison of Suspect and Non-Targeted Screening

The list of features matched with the PMT and KEMI suspect screening lists and the list of features tentatively identified by non-targeted screening were not mutually inclusive. Suspect screening is performed on features with similar MS^1^ monoisotopic mass (±2 mDa) and predicted retention time, whereas the non-targeted screening performed in this study required MS^2^ acquisition. Suspect screening resulted in 26 chemicals from the PMT list and 200 chemicals from the KEMI list matching with features extracted from the recycled textiles. Of these, 56 and 32 features were also prioritized in the non-targeted screening in positive and negative modes, respectively. However, non-targeted screening was able to utilize the data from 768 further features with high-quality MS^2^. Many of these features received confident structural annotations using spectral library matches and *in silico* fragmentation and could be ranked by predicted toxicity and concentration.

Features matched with chemicals on the PMT list and prioritized with suspect screening were rarely observed to rank high in non-targeted screening methods. In positive mode, 2,2′-dimorpholinyldiethyl ether ranked relatively lower (706/990) in non-targeted screening due to its low predicted aquatic toxicity (LC_50_ = 0.47 mM) and concentration (7.9 × 10^–5^ mM). In negative mode, 2,4-dinitrophenol was ranked 50/176 in non-targeted screening with a predicted aquatic LC_50_ of 0.19 mM. 4-aminotoluene-3-sulfonic acid was ranked 168/176 (LC_50_ = 0.73 mM), and dibutyl hydrogen phosphate ranked 162/176 (LC_50_ = 0.62 mM). Only the negative mode feature matched with dinoseb in the PMT list was also highly ranked by non-targeted screening (6/176, LC_50_ = 0.07 mM).

Suspect screening workflows are generally limited by the scope and length of the list of chemicals selected, compared to non-targeted screening where *a priori* chemical information is not required.^13^ By including non-targeted screening, enabled prioritization of features with higher relative predicted toxicities and concentrations compared to suspect screening alone. The highly ranked features reported in this study may have been omitted if non-targeted screening was not performed, providing evidence that this methodology added value to the HRMS acquisition results.

## 4 Conclusions

We have demonstrated, through detection, identification and prioritization of numerous restricted and/or potentially harmful substances, that such chemicals may be present in recycled textile manufacturing and may introduce a risk to human and environmental health. Suspect screening methodologies that employed prioritization lists containing hundreds of chemicals were evaluated against non-targeted screening methods, resulting in comparable lists of features based on their predicted structural annotation, toxicity and concentrations. This study provides a valid methodology for the implementation of suspect and non-targeted screening methodologies that can be used to further investigate chemicals that are detected in textiles and more accurately quantify their concentrations and risk to human and environmental health. For example, the confident detection and identification of dinoterb (Level 2b), a toxic pesticide, detected in 69% of recycled textile samples was prioritized with the application of relative aquatic toxicity predictions using machine learning techniques. Furthermore, industrial chemicals, such as per- and polyfluoroalkyl substances, nitrophenols, organophosphates, polyethylene glycols, and other intermediaries, were detected in recycled textiles intended for extended and close proximity use in humans at concentrations that could be approaching thresholds for adverse toxicological impacts. These discoveries would require further evaluation to assess the risk to end users and any breaches to existing legislation in respective jurisdictions. Furthermore, the fate and transformation of chemicals throughout the recycling process should be investigated to understand the risk more completely. The continued development and manufacture of recycled textiles is an integral part of the principles of the circular economy and will significantly contribute to the reduction of raw materials, waste production and carbon dioxide emissions to the atmosphere. These goals must be met with equal importance to the human and environmental health and should not be the cost of progress.

## Supporting Information Available

Method validation results and acquisition parameters (PDF)
Complete lists of chemicals and features used in calibration, validation and suspect and non-targeted screening (XLSX)

## Notes

MassIVE MSV000095185–mzML converted acquisition files (10.25345/C59C6SC19).

## References

[ref1] Moazzem S.; Crossin E.; Daver F.; Wang L. Assessing environmental impact reduction opportunities through life cycle assessment of apparel products. Sustainable Prod. Consumption 2021, 28, 663–674. 10.1016/j.spc.2021.06.015.

[ref2] Dubois M.; Sims E.; Moerman T.; Watson D.; Bauer B.; Bel J.; Mehlhart G. Guidance for Separate Collection of Municipal Waste; European Commission, Directorate-General for Environment Publications Office: Brussels, Belgium, 2020.

[ref3] Undas A. K.; Groenen M.; Peters R. J. B.; van Leeuwen S. P. J. Safety of recycled plastics and textiles: Review on the detection, identification and safety assessment of contaminants. Chemosphere 2023, 312, 137175 10.1016/j.chemosphere.2022.137175. 36370761

[ref4] Benkhaya S.; M’ rabet S.; El Harfi A. A review on classifications, recent synthesis and applications of textile dyes. Inorg. Chem. Commun. 2020, 115, 107891 10.1016/j.inoche.2020.107891. PMC7002841 32042981

[ref5] Gürses A.; Açıkyıldız M.; Güneş K.; Gürses M. S. Classification of Dye and Pigments. In Dyes and Pigments; Gürses A.; Açıkyıldız M.; Güneş K.; Gürses M. S., Eds.; Springer International Publishing, 2016; pp 31–45.

[ref6] Berradi M.; Hsissou R.; Khudhair M.; Assouag M.; Cherkaoui O.; El Bachiri A.; El Harfi A. Textile finishing dyes and their impact on aquatic environs. Heliyon 2019, 5 (11), e02711 10.1016/j.heliyon.2019.e02711. 31840123 PMC6893069

[ref7] Arslan-Alaton I.; Erdinc E. Effect of photochemical treatment on the biocompatibility of a commercial nonionic surfactant used in the textile industry. Water Res. 2006, 40 (18), 3409–3418. 10.1016/j.watres.2006.07.014. 16978681

[ref8] Holmquist H.; Schellenberger S.; van der Veen I.; Peters G. M.; Leonards P. E. G.; Cousins I. T. Properties, performance and associated hazards of state-of-the-art durable water repellent (DWR) chemistry for textile finishing. Environ. Int. 2016, 91, 251–264. 10.1016/j.envint.2016.02.035. 26994426

[ref9] Rosace G.; Migani V.; Guido E.; Colleoni C. Flame Retardant Finishing for Textiles. In Flame Retardants: Polymer Blends, Composites and Nanocomposites; Visakh P. M.; Arao Y., Eds.; Springer International Publishing, 2015; pp 209–246.

[ref10] Kahoush M.; Kadi N. Towards sustainable textile sector: Fractionation and separation of cotton/ polyester fibers from blended textile waste. Sustainable Mater. Technol. 2022, 34, e00513 10.1016/j.susmat.2022.e00513.

[ref11] Sá M. F.; Castro V.; Gomes A. I.; Morais D. F. S.; Braga R. V. P. S. S.; Saraiva I.; Souza-Chaves B. M.; Park M.; Fernández-Fernández V.; Rodil R.; et al. Tracking pollutants in a municipal sewage network impairing the operation of a wastewater treatment plant. Sci. Total Environ. 2022, 817, 152518 10.1016/j.scitotenv.2021.152518. 34995583

[ref12] Sreedharan V.; Saha P.; Rao K. V. B. Dye degradation potential of Acinetobacter baumannii strain VITVB against commercial azo dyes. Biorem. J. 2021, 25 (4), 347–368. 10.1080/10889868.2020.1871317.

[ref13] Szabo D.; Falconer T. M.; Fisher C. M.; Heise T.; Phillips A. L.; Vas G.; Williams A. J.; Kruve A. Online and Offline Prioritization of Chemicals of Interest in Suspect Screening and Non-targeted Screening with High-Resolution Mass Spectrometry. Anal. Chem. 2024, 96, 3707–3716. 10.1021/acs.analchem.3c05705. 38380899 PMC10918621

[ref14] Bos J. D.; Meinardi M. M. H. M. The 500 Da rule for the skin penetration of chemical compounds and drugs. Exp. Dermatol. 2000, 9 (3), 165–169. 10.1034/j.1600-0625.2000.009003165.x. 10839713

[ref15] Carlsson J.; Iadaresta F.; Eklund J.; Avagyan R.; Östman C.; Nilsson U. Suspect and non-target screening of chemicals in clothing textiles by reversed-phase liquid chromatography/hybrid quadrupole-Orbitrap mass spectrometry. Anal. Bioanal. Chem. 2022, 414 (3), 1403–1413. 10.1007/s00216-021-03766-x. 34786606 PMC8724091

[ref16] Thierse H.-J.; Luch A. Consumer protection and risk assessment: sensitising substances in consumer products. Allergo J. Int. 2019, 28 (6), 167–182. 10.1007/s40629-019-0093-3.

[ref17] Mairinger T.; Loos M.; Hollender J. Characterization of water-soluble synthetic polymeric substances in wastewater using LC-HRMS/MS. Water Res. 2021, 190, 116745 10.1016/j.watres.2020.116745. 33360422

[ref18] Schneider C. A.; Rasband W. S.; Eliceiri K. W. NIH Image to ImageJ: 25 years of image analysis. Nat. Methods 2012, 9 (7), 671–675. 10.1038/nmeth.2089. 22930834 PMC5554542

[ref19] Malm L.; Palm E.; Souihi A.; Plassmann M.; Liigand J.; Kruve A. Guide to Semi-Quantitative Non-Targeted Screening Using LC/ESI/HRMS. Molecules 2021, 26 (12), 3524 10.3390/molecules26123524. 34207787 PMC8228683

[ref20] Taha H. M.; Aalizadeh R.; Alygizakis N.; Antignac J.-P.; Arp H. P. H.; Bade R.; Baker N.; Belova L.; Bijlsma L.; Bolton E. E.; et al. The NORMAN Suspect List Exchange (NORMAN-SLE): facilitating European and worldwide collaboration on suspect screening in high resolution mass spectrometry. Environ. Sci. Eur. 2022, 34 (1), 104 10.1186/s12302-022-00680-6. 36284750 PMC9587084

[ref21] Helmus R.; ter Laak T. L.; van Wezel A. P.; de Voogt P.; Schymanski E. L. patRoon: open source software platform for environmental mass spectrometry based non-target screening. J. Cheminform. 2021, 13 (1), 1 10.1186/s13321-020-00477-w. 33407901 PMC7789171

[ref22] Smith C. A.; Want E. J.; O’Maille G.; Abagyan R.; Siuzdak G. XCMS: Processing Mass Spectrometry Data for Metabolite Profiling Using Nonlinear Peak Alignment, Matching, and Identification. Anal. Chem. 2006, 78 (3), 779–787. 10.1021/ac051437y. 16448051

[ref23] Peter K. T.; Phillips A. L.; Knolhoff A. M.; Gardinali P. R.; Manzano C. A.; Miller K. E.; Pristner M.; Sabourin L.; Sumarah M. W.; Warth B.; Sobus J. R. Nontargeted Analysis Study Reporting Tool: A Framework to Improve Research Transparency and Reproducibility. Anal. Chem. 2021, 93 (41), 13870–13879. 10.1021/acs.analchem.1c02621. 34618419 PMC9408805

[ref24] Gloaguen Y.; Kirwan J. A.; Beule D. Deep Learning-Assisted Peak Curation for Large-Scale LC-MS Metabolomics. Anal. Chem. 2022, 94 (12), 4930–4937. 10.1021/acs.analchem.1c02220. 35290737 PMC8969107

[ref25] Kuhl C.; Tautenhahn R.; Böttcher C.; Larson T. R.; Neumann S. CAMERA: An Integrated Strategy for Compound Spectra Extraction and Annotation of Liquid Chromatography/Mass Spectrometry Data Sets. Anal. Chem. 2012, 84 (1), 283–289. 10.1021/ac202450g. 22111785 PMC3658281

[ref26] Dührkop K.; Fleischauer M.; Ludwig M.; Aksenov A. A.; Melnik A. V.; Meusel M.; Dorrestein P. C.; Rousu J.; Böcker S. SIRIUS 4: a rapid tool for turning tandem mass spectra into metabolite structure information. Nat. Methods 2019, 16 (4), 299–302. 10.1038/s41592-019-0344-8. 30886413

[ref27] Horai H.; Arita M.; Kanaya S.; Nihei Y.; Ikeda T.; Suwa K.; Ojima Y.; Tanaka K.; Tanaka S.; Aoshima K.; et al. MassBank: a public repository for sharing mass spectral data for life sciences. J. Mass Spectrom. 2010, 45 (7), 703–714. 10.1002/jms.1777. 20623627

[ref28] Ruttkies C.; Schymanski E. L.; Wolf S.; Hollender J.; Neumann S. MetFrag relaunched: incorporating strategies beyond in silico fragmentation. J. Cheminform. 2016, 8 (1), 3 10.1186/s13321-016-0115-9. 26834843 PMC4732001

[ref29] Schymanski E. L.; Jeon J.; Gulde R.; Fenner K.; Ruff M.; Singer H. P.; Hollender J. Identifying Small Molecules via High Resolution Mass Spectrometry: Communicating Confidence. Environ. Sci. Technol. 2014, 48 (4), 2097–2098. 10.1021/es5002105. 24476540

[ref30] Arp H. P. H.; Hale S. E.; Schliebner I.; Neumann M. Prioritised PMT/vPvM Substances in the REACH Registration Database; Dessau-Roßlau Germany, 2023 https://www.umweltbundesamt.de/en/publikationen/prioritised-pmtvpvm-substances-in-the-reach.

[ref31] Aalizadeh R.; Alygizakis N. A.; Schymanski E. L.; Krauss M.; Schulze T.; Ibáñez M.; McEachran A. D.; Chao A.; Williams A. J.; Gago-Ferrero P.; et al. Development and Application of Liquid Chromatographic Retention Time Indices in HRMS-Based Suspect and Nontarget Screening. Anal. Chem. 2021, 93 (33), 11601–11611. 10.1021/acs.analchem.1c02348. 34382770

[ref32] Sepman H.; Malm L.; Peets P.; MacLeod M.; Martin J.; Breitholtz M.; Kruve A. Bypassing the Identification: MS2Quant for Concentration Estimations of Chemicals Detected with Nontarget LC-HRMS from MS2 Data. Anal. Chem. 2023, 95 (33), 12329–12338. 10.1021/acs.analchem.3c01744. 37548594 PMC10448440

[ref33] Peets P.; Wang W.-C.; MacLeod M.; Breitholtz M.; Martin J. W.; Kruve A. MS2Tox Machine Learning Tool for Predicting the Ecotoxicity of Unidentified Chemicals in Water by Nontarget LC-HRMS. Environ. Sci. Technol. 2022, 56, 15508 10.1021/acs.est.2c02536. 36269851 PMC9670854

[ref34] Fisher C. M.; Peter K. T.; Newton S. R.; Schaub A. J.; Sobus J. R. Approaches for assessing performance of high-resolution mass spectrometry–based non-targeted analysis methods. Anal. Bioanal. Chem. 2022, 414 (22), 6455–6471. 10.1007/s00216-022-04203-3. 35796784 PMC9411239

[ref35] Elapavalore A.; Kondić T.; Singh R. R.; Shoemaker B. A.; Thiessen P. A.; Zhang J.; Bolton E. E.; Schymanski E. L. Adding open spectral data to MassBank and PubChem using open source tools to support non-targeted exposomics of mixtures. Environ. Sci.: Process. Impacts 2023, 25 (11), 1788–1801. 10.1039/D3EM00181D. 37431591 PMC10648001

[ref36] United States Environmental Protection Agency (USEPA). Chemical Data Reporting Data (ChemView), 2020. https://www.epa.gov/chemical-data-reporting. (accessed March 2024).

[ref37] Malnes D.; Ahrens L.; Köhler S.; Forsberg M.; Golovko O. Occurrence and mass flows of contaminants of emerging concern (CECs) in Sweden’s three largest lakes and associated rivers. Chemosphere 2022, 294, 133825 10.1016/j.chemosphere.2022.133825. 35114267

[ref38] Gago-Ferrero P.; Krettek A.; Fischer S.; Wiberg K.; Ahrens L. Suspect Screening and Regulatory Databases: A Powerful Combination To Identify Emerging Micropollutants. Environ. Sci. Technol. 2018, 52 (12), 6881–6894. 10.1021/acs.est.7b06598. 29782800

[ref39] Heusinkveld H. J.; van Vliet A. C.; Nijssen P. C. G.; Westerink R. H. S. In vitro neurotoxic hazard characterisation of dinitrophenolic herbicides. Toxicol. Lett. 2016, 252, 62–69. 10.1016/j.toxlet.2016.04.014. 27106277

[ref40] Yao C.; Yang H.; Li Y. A review on organophosphate flame retardants in the environment: Occurrence, accumulation, metabolism and toxicity. Sci. Total Environ. 2021, 795, 148837 10.1016/j.scitotenv.2021.148837. 34246143

[ref41] Mäkinen M. S. E.; Mäkinen M. R. A.; Koistinen J. T. B.; Pasanen A.-L.; Pasanen P. O.; Kalliokoski P. J.; Korpi A. M. Respiratory and Dermal Exposure to Organophosphorus Flame Retardants and Tetrabromobisphenol A at Five Work Environments. Environ. Sci. Technol. 2009, 43 (3), 941–947. 10.1021/es802593t. 19245040

[ref42] Cristale J.; Santos I. O.; de Aragão Umbuzeiro G.; Fagnani E. Occurrence and risk assessment of organophosphate esters in urban rivers from Piracicaba watershed (Brazil). Environ. Sci. Pollut. Res. 2021, 28 (42), 59244–59255. 10.1007/s11356-020-10150-2. 32748359

[ref43] Fu L.; Bin L.; Cui J.; Nyobe D.; Li P.; Huang S.; Fu F.; Tang B. Tracing the occurrence of organophosphate ester along the river flow path and textile wastewater treatment processes by using dissolved organic matters as an indicator. Sci. Total Environ. 2020, 722, 137895 10.1016/j.scitotenv.2020.137895. 32208263

[ref44] Kojima H.; Takeuchi S.; Itoh T.; Iida M.; Kobayashi S.; Yoshida T. In vitro endocrine disruption potential of organophosphate flame retardants via human nuclear receptors. Toxicology 2013, 314 (1), 76–83. 10.1016/j.tox.2013.09.004. 24051214

[ref45] Azizi S.; Dehghani M. H.; Naddafi K.; Nabizadeh R.; Yunesian M. Occurrence of organophosphorus esters in outdoor air fine particulate matter and comprehensive assessment of human exposure: A global systematic review. Environ. Pollut. 2023, 318, 120895 10.1016/j.envpol.2022.120895. 36529340

[ref46] Gago-Ferrero P.; Bletsou A. A.; Damalas D. E.; Aalizadeh R.; Alygizakis N. A.; Singer H. P.; Hollender J.; Thomaidis N. S. Wide-scope target screening of > 2000 emerging contaminants in wastewater samples with UPLC-Q-ToF-HRMS/MS and smart evaluation of its performance through the validation of 195 selected representative analytes. J. Hazard. Mater. 2020, 387, 121712 10.1016/j.jhazmat.2019.121712. 31784138

[ref47] Barani H.; Montazer M. A Review on Applications of Liposomes in Textile Processing. J. Liposome Res. 2008, 18 (3), 249–262. 10.1080/08982100802354665. 18770074

[ref48] Singha K.; Pandit P.; Maity S.; Sharma S. R. Harmful Environmental Effects for Textile Chemical Dyeing Practice. In Green Chemistry for Sustainable Textiles; Ibrahim N.; Hussain C.; M, Eds.; Woodhead Publishing, 2021; pp 153–164.

[ref49] Das A.; Baidya R.; Chakraborty T.; Samanta A. K.; Roy S. Pharmacological basis and new insights of taxifolin: A comprehensive review. Biomed. Pharmacother. 2021, 142, 112004 10.1016/j.biopha.2021.112004. 34388527

[ref50] Kanwal Q.; Hussain I.; Siddiqui H. L.; Javaid A. Antifungal activity of flavonoids isolated from mango (*Mangifera indica* L.) leaves. Nat. Prod. Res. 2010, 24 (20), 1907–1914. 10.1080/14786419.2010.488628. 21108117

[ref51] Secim-Karakaya P.; Saglam-Metiner P.; Yesil-Celiktas O. Antimicrobial and wound healing properties of cotton fabrics functionalized with oil-in-water emulsions containing Pinus brutia bark extract and Pycnogenol for biomedical applications. Cytotechnology 2021, 73 (3), 423–431. 10.1007/s10616-021-00467-2. 34149175 PMC8167011

[ref52] Ju K.-S.; Parales Rebecca E. Nitroaromatic Compounds, from Synthesis to Biodegradation. Microbiol. Mol. Biol. Rev. 2010, 74 (2), 250–272. 10.1128/mmbr.00006-10. 20508249 PMC2884413

[ref53] van der Oost R.; Beyer J.; Vermeulen N. P. E. Fish bioaccumulation and biomarkers in environmental risk assessment: a review. Environ. Toxicol. Pharmacol. 2003, 13 (2), 57–149. 10.1016/S1382-6689(02)00126-6. 21782649

