# Supplementary material for: Prioritization, Identification, and Quantification of Emerging Contaminants in Recycled Textiles Using Non-Targeted and Suspect Screening Workflows by LC-ESI-HRMS
Source: Anal Chem. 2024 Aug 20;96(35):14150–9. doi: 10.1021/acs.analchem.4c02041 (PMC11375621; doi:10.1021/acs.analchem.4c02041)
Supplement: Supplementary file 1 — ac4c02041_si_001.pdf [file ac4c02041_si_001.pdf]

SUPPORTING INFORMATION

Prioritisation, Identification, and Semi-Quantification of Emerging Contaminants in Textiles Using Non Target and Suspect Screening Workflows by LC-ESI-HRMS

Drew Szabo[1], Stellan Fischer[2], Aji P. Mathew[1], Anneli Kruve[1*]

[1]Department of Materials and Environmental Chemistry (MMK), Stockholm University, Svante Arrhenius Väg 16, SE-11418 Stockholm, Sweden

[2]Swedish Chemicals Agency (Kemi), SE-17267 Stockholm, Sweden

Contents

Textile Extraction Solvent Optimization S2

Extraction Evaluation and Performance S3

Workflow Parameters S5

6 Pages, 3 Figures, 3 Tables; 8 additional tables in .xls

Textile Extraction Solvent Optimization

Textile samples were selected idependantly from the study and were comprised of 100% virgin cotton. Triplicate samples of 1 x 1 cm were weighed and imaged under 55x magnification to determine the density (grams per square meter, GSM) and thread count (warps and wefts per square centimeter) (Table S8).

Table S9: Summary of density and thread count characteristics for cotton textile sample.

Textile	Density (g m^{-2}; GSM)	Thread count (count cm^{-2})
100% Cotton	339 ± 29	weft: 16 ± 0.82<br>warp: 11 ± 0.47

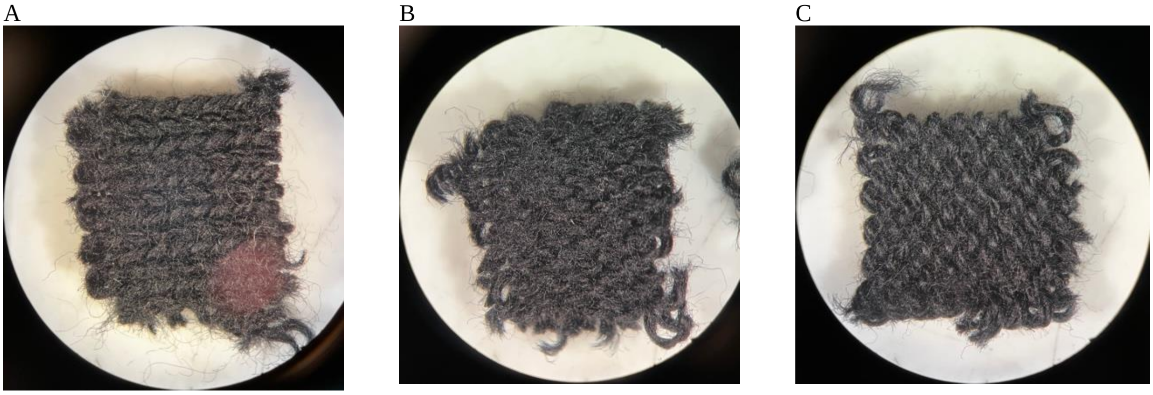

Figure S1: Magnified images (55x) of triplicate cotton textile samples for thread count characterisation.

A multi-residue approach was tested to extract the highest number of detectable organic contaminatns from the textiles using different proportions of methanol and acetonitrile. The solvents selected for extraction were: i) methanol, ii) 1:1 methanol & acetonitrile, and iii) acetonitrile. Breifly, 5 mL solvent was added to a 15 mL glass vial containing 1 cm^2 textile sample and sonicated for 30 minutes. The supernatant was decanted to a new 15 mL glass vial and the extraction was repeated with a further 5 mL solven and 30 minute sonication. The supernatants from both extractions were combined and evaporated to dryness at room temperature under a stream of nitrogen gas. 1.00 mL of solvent was added to each vial to reconstitute the mixure and was filtered (0.20 µm, PES) into a 1 mL glass autosampling vial

for analysis. Acquisition and data processing was performed using method described in Section 2.3 and 2.4 resepctively.

Extraction Evaluation and Performance

The total number of features were evaluated for each of the extraction solvents: methanol, acetonitrile and a 1:1 mixture. A total of 4,936 unique features were detected using all solvents, where 2,298 were detected with methanol, 2,612 were detected with acetonitrile, and 3,519 were detected with the mixture. 908 features were common between the extraction solvents,

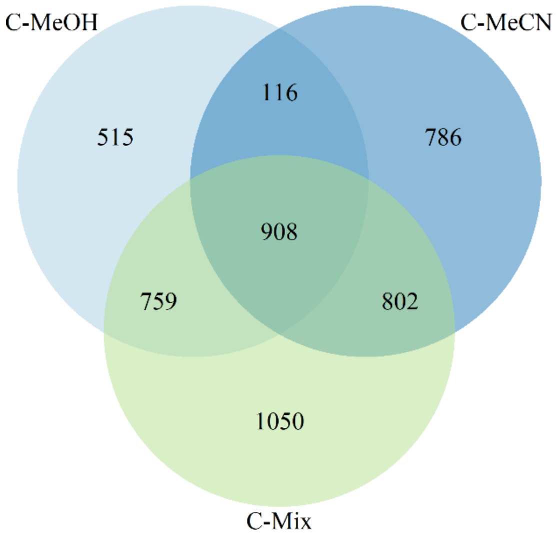

Figure S2: Number of features detected from textiles extracted with methanol (MeOH), acetonitrile (MeCN) and an equal mixture.

The majority of the 4,936 features with extractable MS^1 peaks were distributed between 100 – 1,000 *m/z* for all solvent types. Furthermore, most of the extractable features were eluted from the column throughout the 25 minute run time as the organic phase was increased to 100% (Figure S3-A). There were more chemicals with mass <500 Da that were extracted by methanol, compared with the mixture and acetonitrile. However there were more chemicals with mass >500 Da that were extracted with acetonitrile. Similarly, methanol provided greater extraction for chemicals with earlier eluting times, and acetonitrile had greater extraction for

late eluting chemicals. The mixture provided a balanced extraction of chemicals from high and low *m/z* and RT.

A total of 1,957 features had MS^2 acquired and were structurally annotated by SIRIUS: CSI FingerID. The predicted hydrophobicity of each feature was then calculated and was distributed around a mean of 5 log units (Figure S3-B). Methanol was more effective at extracting chemicals with log*P* between 0 – 10, however, acetonitrile and the mixture were more effective at extracting chemicals with log*P* > 10. Again, the mixture provided a balanced extraction solvent compared to using methanol or acetonitrile individually.

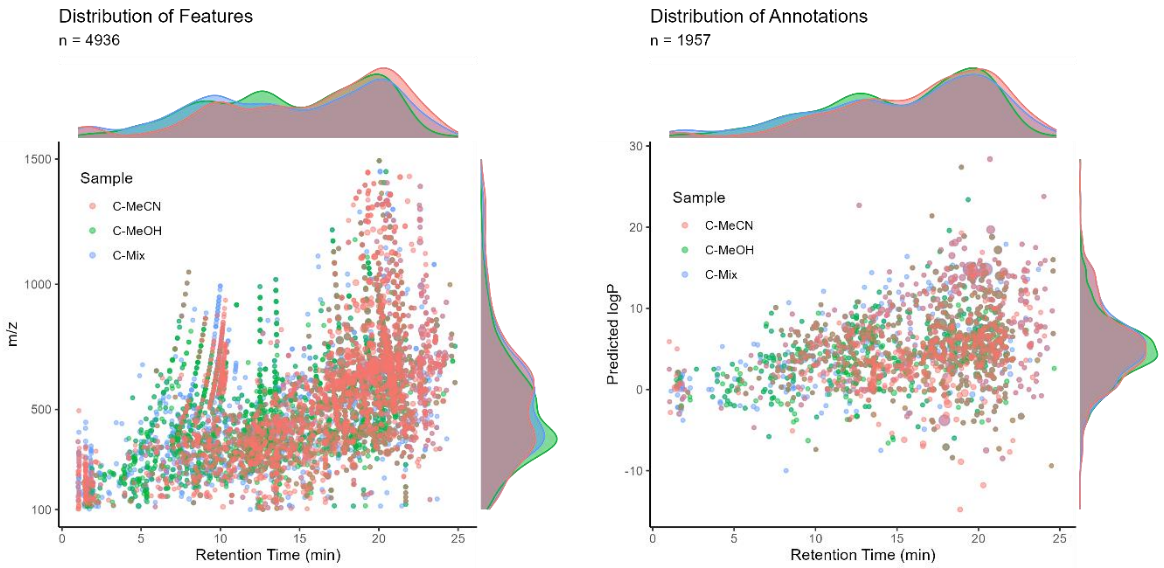

Figure S3: Distribution of total features (left) and features annotated by SIRIUS CSI:FingerID (right) for cotton textile sample extracted with methanol (MeOH), acetonitrile (MeCN) and a mixture.

The mixture of methanol and acetonitrile was selected to perform the extractions of organic chemicals from recycled textile samples in this study. The selection of solvents is far from a complete extraction optimisation, further investigation is required to optimise the ratio of solvents, and other parameters such as the temperature and duration. This methodology provided a safe and effective extraction methodology that requires no clean up or filtering to ensure little to no chemical loss during the process.

Workflow Parameters

XCMS was used for peak picking using the *CentWave* algorithm, then grouping was performed with the *PeakDensity* and *Obiwarp* functions respectively. mzR was then used to perform peak extraction of each feature, where the average MS^1 and MS^2 peak lists were used for structural annotation (Table S9).

Table S10: Parameters used in peak picking and grouping with XCMS, and peak extraction with mzR packages.

**Parameter**	**Value**
CentWave	
ppm	5
peakwidth	10 – 60
snthresh	3
prefilter	3, 100
mzCenterFun	wMean
intergrate	1
mzdiff	0.005
Noise	1000
PeakDensity	
minFraction	0
minSamples	1
bw	15
binSize	0.01
Obiwarp	
response	1
gapInit	0.3
gapExtend	2.4
factorDiag	2
factorGap	1
binSize	0.05
mzR	
maxMSRtWindow	5
precursorMzWindow	0.2
clusterMzWindow	0.005
topmost	250
maxIsotopes	4
mzDefectRange	-0.1 – 0.1

Structural annotation of features with acquired MS^2 were matched with the MassBank library, and predicted using MetFrag and SIRIUS. MS^1 error were kept the same between each algorithm to provide consistency in analysis. The MS^2 error is measured in ppm for SIRIUS and then mDa for MetFrag and library matches. Only the top 5 compounds were computed and saved, where the top 1 compound was filtered for the final analysis (Table S10).

Table S11: Parameters used for structural annotation using library and in silico tools SIRIUS and MetFrag.

**Parameter**	*SIRIUS*	*MetFrag*	*Library*
Library	PubChem	PubChemLite	MassBank
MS1 error	5 ppm	5 ppm	5 ppm
MS2 error	50 ppm	20 mDa	20 mDa
cores	4	NA	NA
topMost	5	5	5
elements	CHONPSFClBr	NA	NA
maxCandidatesToStop	NA	2500	NA
